# A Longitudinal and Multimodal Simulation-Based Curriculum Improved Internal Medicine Residents’ Confidence in Procedures, Point-of-Care Ultrasound, and Clinical Decision-Making

**DOI:** 10.7759/cureus.98713

**Published:** 2025-12-08

**Authors:** Sarah Grebennikov, Aisha Imam, Megan Kang, Aimee E Willett, Brad Gable, Sanjay Patel

**Affiliations:** 1 Internal Medicine, OhioHealth Riverside Methodist Hospital, Columbus, USA; 2 Emergency Medicine, OhioHealth Riverside Methodist Hospital, Columbus, USA

**Keywords:** curriculum development and evaluation, graduate medical education (gme), innovation in medical education, internal medicine residency training, point-of-care ultrasound (pocus), procedural skills training, simulation-based education (sbe)

## Abstract

Introduction

Internal medicine training has historically relied on classroom-based lectures, typically delivered through morning and noon conferences, which offer broad content coverage but limited experiential learning. Simulation-based education improves resident confidence, procedural ability, and clinical reasoning; yet it is often delivered in brief, standalone activities that lack longitudinal structure or integration within the broader curriculum. Few programs have a longitudinal simulation curriculum that spans the academic year and incorporates multiple modalities and topics.

We developed a novel simulation-based curriculum intentionally integrated into the existing resident academic schedule to provide structured hands-on learning across multiple domains. Sessions focused on procedural and point-of-care ultrasound (POCUS) training, as well as separate high-fidelity clinical cases. The curriculum includes four fundamental procedures: POCUS instruction on cardiac, pulmonary, abdominal, genitourinary, and vascular applications, and 14 high-fidelity clinical triage cases. In total, 50 of approximately 150 annual didactic hours were redesigned into simulation-based sessions.

Materials and methods

The longitudinal curriculum spanned nine months (August 2024 to April 2025) and was divided into two components: procedural and POCUS training, and clinical simulation scenarios. Procedural training included central venous catheterization, thoracentesis, intubation, and paracentesis. POCUS training included cardiac, pulmonary, abdominal/genitourinary, and vascular ultrasound. Four residents were assigned to the procedural station and another four to the ultrasound station, with groups rotating between stations every 30 minutes. Eight residents were assigned to clinical scenarios that ran concurrently, consisting of a 10-minute simulation followed by a 20-minute debrief.

Outcomes

The Phillips return on investment (ROI) framework (levels 0-3) guided evaluation through post-session resident surveys and an end-of-year faculty survey assessing curriculum effectiveness. Outcomes were analyzed using mean Likert scores and response frequencies.

Results

Thirty-six first-year residents participated. A total of 288 surveys were collected: 89 for procedures, 87 for POCUS, and 112 for clinical simulations. Across all domains, 284 of 288 responses (98.6%) agreed or strongly agreed that the sessions provided new information or clarified existing knowledge.

Specific findings included the following: all participants (89/89) agreed or strongly agreed that they felt more confident in their procedural ability; 98.9% (86/87) agreed or strongly agreed that they were more confident in distinguishing normal from abnormal sonographic images; and 94.7% (106/112) agreed or strongly agreed that clinical simulation improved their diagnostic skills.

Five critical care attendings who supervised interns throughout the year reported observable improvements compared to prior cohorts. All five noted stronger POCUS skills, and four reported improved triaging skills. Both improvements were attributed to the simulation curriculum.

Conclusions

A longitudinal simulation curriculum that integrates POCUS, procedures, and high-fidelity clinical cases improves resident confidence, procedural competence, and diagnostic decision-making. This multimodal model offers a scalable approach for strengthening experiential learning in internal medicine training.

## Introduction

Graduate internal medicine education traditionally relies on morning and noon lectures to teach core concepts [1]. At our 1000-bed teaching hospital, we train 18 categorical internal medicine residents per year in a three-year program, along with six preliminary and 12 transitional year residents. The curriculum typically includes around 150 annual noon conferences covering key topics and case discussions, reflecting the traditional lecture-based structure used in many internal medicine programs [1].

Although this format provides broad content exposure, it offers limited opportunities for experiential learning and practical skill development [2,3]. Prior studies have demonstrated that simulation-based education improves diagnostic accuracy, procedural proficiency, team communication, and confidence in managing acutely ill patients compared to traditional lecture-based learning [4-7]. Despite these benefits, simulation within internal medicine training is not routinely integrated and often remains fragmented, limited to orientation events, isolated procedural checkoffs, or brief exposure sessions without longitudinal reinforcement [8-11]. Few internal medicine programs have incorporated simulation as a longitudinal and central component of their didactic schedule, and even fewer have comprehensively evaluated the outcomes of such curricular redesigns [9,11-13]. Even when simulation is delivered longitudinally, it rarely integrates multiple modalities and topics, such as procedure training, point-of-care ultrasound (POCUS), and high-fidelity clinical cases, into a coordinated framework [14,15]. Furthermore, no published studies to date have systematically examined the impact of a simulation-based curriculum as a substitute for didactic lectures in internal medicine residency education [4].

To address this gap, we implemented a comprehensive simulation-based curriculum that replaced one-third of traditional lecture sessions with experiential learning in procedural skills, POCUS, and acute clinical decision-making. The objective of this study was to evaluate the impact of this curricular transition on the confidence and competence of internal medicine residents in procedures, point-of-care ultrasound, and patient triage. Resident confidence was measured using self-reported surveys, and resident competence in procedures, POCUS, and patient care was assessed through faculty surveys.

## Materials and methods

Curriculum design

The curriculum was developed by the chief residents in collaboration with faculty leadership. Planning began five months prior to implementation, and the curriculum ran from August 2024 to April 2025. It was developed in response to feedback from incoming and current residents who requested additional simulation training, as well as program concerns about gaps in procedural skills and acute care triage identified during prior assessments. The educational approach emphasized hands-on experiential learning, repeated skills exposure, case-based decision-making, and structured debriefing after each session. Sessions were organized by system-based themes: cardiac, pulmonary, abdominal, and vascular. POCUS and procedure topics were determined by internal medicine faculty based on procedures residents commonly perform and ultrasound topics with direct bedside application. Clinical case topics were created by the chief residents in collaboration with internal medicine faculty to reflect common and high-stakes inpatient scenarios. While formal validation studies were not conducted, this represents a pragmatic, needs-based approach appropriate for early-phase curriculum implementation. Each body system included five one-hour sessions that alternated between clinical simulations and combined procedural and POCUS training.

Curriculum structure

**Figure 1:**
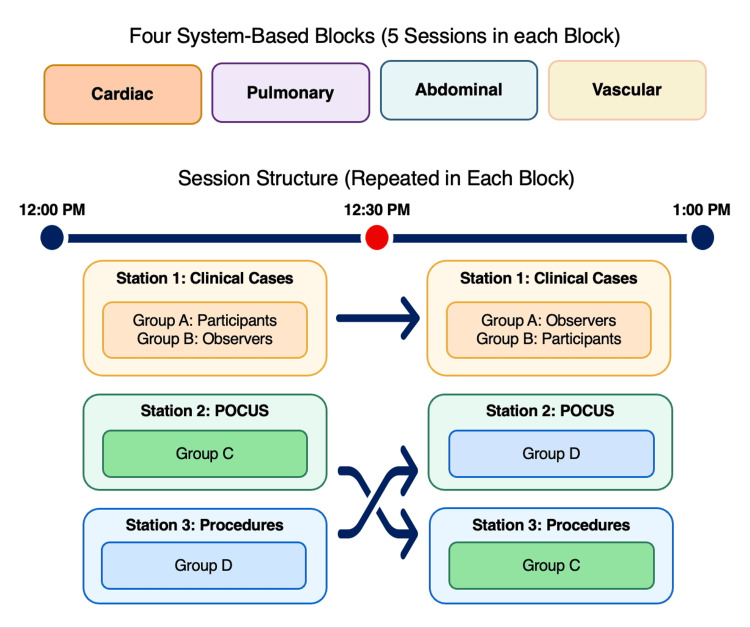
Simulation curriculum structure and session rotation design. POCUS: point-of-care ultrasound.

The simulation curriculum was organized into four system-based blocks: cardiac, pulmonary, abdominal, and vascular. Each block consisted of five noon conference sessions delivered on separate days throughout the academic year. Residents were pre-assigned to specific session dates and groups at the start of the year, ensuring that every post-graduate year (PGY)-1 resident completed both POCUS/procedures and clinical scenarios within each system-based block. In each one-hour session, 16 PGY-1 residents were divided into four groups of four (Groups A through D). Groups A and B were assigned to the clinical simulation station, and Groups C and D were assigned to the POCUS and procedural skills stations (Figure 1).

For simulation cases, four residents actively participated while four observed. During the first half of the session, Group A managed the case, and Group B observed; during the second half, they exchanged roles so that Group B was active and Group A observed. Each scenario lasted approximately 10 minutes, followed by a 20-minute faculty-led debrief, ensuring that all residents experienced both direct participation and observation. A total of 14 clinical case topics were developed, encompassing a range of low-frequency but high-risk scenarios, including ventricular storm and hyponatremic seizures. Topics also covered commonly encountered inpatient problems, such as atrial fibrillation with rapid ventricular response, heart block, cardiogenic shock, ventricular tachycardia, respiratory failure, gastrointestinal bleeding, and goals-of-care conversations.

Groups C and D rotated between the POCUS and procedural skills stations, which followed a similar rotational structure. During the first 30 minutes of the session, Group C completed POCUS training while Group D practiced procedures; after 30 minutes, the groups switched stations so that each resident spent half of the session in POCUS and half in procedural skills training. The 30-minute interval was selected to fit within the noon conference hour while still providing sufficient time for focused hands-on practice in each domain and to maintain learner engagement by limiting continuous time at a single station. Procedural training included central venous catheterization (CVC), thoracentesis, paracentesis, and intubation. POCUS sessions focused on cardiac, lung, abdominal, and vascular ultrasound imaging. Feedback was provided immediately after each station by the faculty.

Participants

The program included 36 PGY-1 residents, including 12 preliminary medicine and six transitional year residents. The curriculum was specifically targeted to PGY-1 residents based on logistical constraints related to scheduling and simulation lab availability. The curriculum was also designed to ensure all PGY-1 residents completed all stations in each system-based block over the academic year.

Facilitation

The clinical case simulations were led by the internal medicine chief residents in collaboration with three simulation specialists, who assisted with mannequin operation and acted as nursing confederates in the scenarios. The chief residents completed a course on experiential learning and debriefing prior to facilitating the pre-brief and debrief of each session. The POCUS and procedure sessions were led by internal medicine faculty as well as pulmonary and critical care attendings.

Measurements

We evaluated our educational intervention using our standardized return on investment in learning (ROL) methodology, which is based on the Phillips return on investment (ROI) model. In this study, we assessed ROL levels 0 through 3 of a possible five levels: level 0 (participation count); level 1 (learner reaction, including relevance and satisfaction); level 2 (learning, including self-reported knowledge and confidence); and level 3 (application, based on faculty-observed clinical performance) [16].

Residents completed post-session surveys immediately after each simulation-based educational activity. Survey questions used retrospective self-assessment language to capture perceived changes in knowledge and skills following each session. The survey has not been previously validated; however, it follows the Phillips ROI method and uses a five-point Likert scale to evaluate session content, facilitation, learning environment, and self-assessed confidence in clinical objectives [16]. Faculty were surveyed at the end of the year to evaluate changes in intern performance.

Data acquisition and analysis

Survey results were collected and managed using REDCap electronic data capture tools (Vanderbilt University, Nashville, TN) hosted at OhioHealth [17]. De-identified responses were then exported to Microsoft Excel (Microsoft Corporation, Redmond, WA). Analysis was done by study investigators using Jamovi v2.6 (retrieved from https://www.jamovi.org). Because this study represented an early implementation and feasibility evaluation, no inferential testing was performed. Formal blinding was not implemented, given the program evaluation nature of the study; however, resident self-reported data were supplemented with independent faculty assessments to provide external validation.

Ethical considerations

This project was reviewed by the OhioHealth Institutional Review Board and was determined to be exempt as a quality improvement initiative (IRB No. FY25-103).

## Results

Thirty-six PGY-1 internal medicine residents completed the curriculum. A cumulative total of 288 surveys was collected, including 89 survey responses for procedures, 87 for POCUS, and 112 for clinical simulations.

Across all domains, between 95.6% and 100% of residents agreed or strongly agreed that the content was relevant, facilitators were knowledgeable, and the learning environment supported their development. Residents consistently reported increased confidence in procedural steps, ultrasound acquisition, and acute clinical management (Tables 1-3).

**Table 1:** Procedure training return on investment and objective survey results (N = 89).

Survey question	"Strongly agree"	"Agree"	"Neutral"	"Disagree"	"Strongly disagree"	Likert scale ± standard deviation
	5	4	3	2	1	(1-5)
The content was relevant to my work	73.0% (65/89)	25.8% (23/89)	1.1% (1/89)	0.0% (0/89)	0.0% (0/89)	4.72 ± 0.47
This training provided me with new information (or clarified existing information)	75.3% (67/89)	23.6% (21/89)	1.1% (1/89)	0.0% (0/89)	0.0% (0/89)	4.74 ± 0.46
I intend to use what I learned from this training	76.4% (68/89)	22.5% (20/89)	1.1% (1/89)	0.0% (0/89)	0.0% (0/89)	4.75 ± 0.46
The facilitator(s) was knowledgeable about the subject	82.0% (73/89)	18.0% (16/89)	0.0% (0/89)	0.0% (0/89)	0.0% (0/89)	4.82 ± 0.38
The facilitator(s) was effective in helping me learn new information (or clarify existing information)	79.8% (71/89)	20.2% (18/89)	0.0% (0/89)	0.0% (0/89)	0.0% (0/89)	4.80 ± 0.40
The facilitator(s) was responsive to participants' needs and questions	82.0% (73/89)	18.0% (16/89)	0.0% (0/89)	0.0% (0/89)	0.0% (0/89)	4.82 ± 0.38
The learning environment was conducive to learning	80.9% (72/89)	19.1% (17/89)	0.0% (0/89)	0.0% (0/89)	0.0% (0/89)	4.81 ± 0.39
I am more confident in my ability to explain the indications and contraindications of the procedure	58.4% (52/89)	38.2% (34/89)	3.4% (3/89)	0.0% (0/89)	0.0% (0/89)	4.56 ± 0.63
I am more confident in my ability to identify relevant anatomy using appropriate adjuncts	60.7% (54/89)	39.3% (35/89)	0.0% (0/89)	0.0% (0/89)	0.0% (0/89)	4.62 ± 0.49
I am more confident in my ability to demonstrate the procedural steps	58.4% (52/89)	41.6% (37/89)	0.0% (0/89)	0.0% (0/89)	0.0% (0/89)	4.58 ± 0.49

**Table 2:** Point-of-care ultrasound return on investment and objective survey results (n = 87).

Survey question	"Strongly agree"	"Agree"	"Neutral"	"Disagree"	"Strongly disagree"	Likert scale ± standard deviation
	5	4	3	2	1	(1-5)
The content was relevant to my work	73.6% (64/87)	26.4% (23/87)	0.0% (0/87)	0.0% (0/87)	0.0% (0/87)	4.74 ± 0.44
This training provided me with new information (or clarified existing information)	74.7% (65/87)	24.1% (21/87)	1.1% (1/87)	0.0% (0/87)	0.0% (0/87)	4.74 ± 0.47
I intend to use what I learned from this training	77.0% (67/87)	23.0% (20/87)	0.0% (0/87)	0.0% (0/87)	0.0% (0/87)	4.77 ± 0.42
The facilitator(s) was knowledgeable about the subject	78.2% (68/87)	21.8% (19/87)	0.0% (0/87)	0.0% (0/87)	0.0% (0/87)	4.78 ± 0.41
The facilitator(s) was effective in helping me learn new information (or clarify existing information)	77.0% (67/87)	23.0% (20/87)	0.0% (0/87)	0.0% (0/87)	0.0% (0/87)	4.77 ± 0.42
The facilitator(s) was responsive to participants' needs and questions	79.3% (69/87)	20.7% (18/87)	0.0% (0/87)	0.0% (0/87)	0.0% (0/87)	4.79 ± 0.41
The learning environment was conducive to learning	78.2% (68/87)	20.7% (20/87)	1.1% (1/87)	0.0% (0/87)	0.0% (0/87)	4.77 ± 0.45
I am more confident in my ability to define the sonographic anatomy	55.2% (48/87)	42.5% (37/87)	2.3% (2/87)	0.0% (0/87)	0.0% (0/87)	4.53 ± 0.54
I am more confident in my ability to obtain sonographic images	56.3% (49/87)	43.7% (38/87)	0.0% (0/87)	0.0% (0/87)	0.0% (0/87)	4.56 ± 0.50
I am more confident in my ability to differentiate between normal and abnormal images	52.9% (46/87)	46.0% (40/87)	1.1% (1/87)	0.0% (0/87)	0.0% (0/87)	4.52 ± 0.52

**Table 3:** Clinical cases' return on investment and objective results (N = 112).

Survey question	"Strongly agree"	"Agree"	"Neutral"	"Disagree"	"Strongly disagree"	Likert scale ± standard deviation
	5	4	3	2	1	(1-5)
The content was relevant to my work	68.8% (77/112)	30.4% (34/112)	0.9% (1/112)	0.0% (0/112)	0.0% (0/112)	4.68 ± 0.49
This training provided me with new information (or clarified existing information)	61.6% (69/112)	35.7% (40/112)	1.8% (2/112)	0.9% (1/112)	0.0% (0/112)	4.58 ± 0.58
I intend to use what I learned from this training	68.8% (77/112)	26.8% (30/112)	4.5% (5/112)	0.0% (0/112)	0.0% (0/112)	4.64 ± 0.56
The facilitator(s) was knowledgeable about the subject	73.2% (82/112)	25.9% (29/112)	0.9% (1/112)	0.0% (0/112)	0.0% (0/112)	4.72 ± 0.47
The facilitator(s) was effective in helping me learn new information (or clarify existing information)	71.4% (80/112)	27.7% (31/112)	0.9% (1/112)	0.0% (0/112)	0.0% (0/112)	4.71 ± 0.48
The facilitator(s) was responsive to participants' needs and questions	71.4% (80/112)	28.6% (32/112)	0.0% (0/112)	0.0% (0/112)	0.0% (0/112)	4.71 ± 0.45
The learning environment was conducive to learning	69.6% (78/112)	29.5% (33/112)	0.9% (1/112)	0.0% (0/112)	0.0% (0/112)	4.69 ± 0.48
I am more confident in my ability to recognize and diagnose a clinical condition	50.9% (57/112)	43.8% (49/112)	4.5% (5/112)	0.9% (1/112)	0.0% (0/112)	4.45 ± 0.62
I am more confident in my ability to manage and stabilize a clinical condition	52.7% (59/112)	41.1% (46/112)	5.4% (6/112)	0.9% (1/112)	0.0% (0/112)	4.46 ± 0.64
I am more confident in my ability to communicate and work in a team	54.5% (61/112)	41.1% (46/112)	3.6% (4/112)	0.9% (1/112)	0.0% (0/112)	4.49 ± 0.61

Procedural training results

A total of 89 responses were collected following procedural simulation sessions, which included CVC, thoracentesis, paracentesis, and endotracheal intubation. Across all domains of instructional delivery, the feedback was highly favorable. Specifically, 98.8% of respondents either strongly agreed or agreed that the training content was relevant to their clinical responsibilities. Of the respondents, 100% indicated that facilitators were knowledgeable and responsive, and that the learning environment was conducive to skill acquisition (Table 1).

Regarding skill-based outcomes, 96.6% of residents reported increased confidence in explaining indications and contraindications for procedures. Of the respondents, 100% reported improved confidence in acquiring relevant sonographic anatomy and executing the procedural steps (Table 1). These results reflect a strong translation of didactic instruction into psychomotor skill development. Qualitative comments from residents emphasized the value of hands-on practice, direct faculty feedback, and the opportunity to practice low-frequency procedures in a supportive, non-evaluative environment.

Point-of-care ultrasound (POCUS) training results

Eighty-seven responses were collected for POCUS training sessions covering cardiac, pulmonary, abdominal, and vascular assessments. Residents overwhelmingly endorsed the training’s relevance and quality, with 100% agreeing or strongly agreeing that the content was clinically applicable and well facilitated (Table 2). Facilitators received high marks across all domains, including knowledge, communication, and responsiveness to learner needs.

In terms of perceived skill gains, 97.7% of residents reported increased confidence in defining sonographic anatomy, 100% in image acquisition, and 98.8% in differentiating normal from abnormal findings. Several residents indicated a desire for more frequent POCUS opportunities and suggested that timing sessions before intensive care unit rotations could enhance clinical readiness. Some respondents also noted the importance of longitudinal exposure for retention of these foundational skills. These findings suggest that even a brief, focused curriculum can substantially improve core ultrasound competencies among interns.

Clinical simulation case results

The clinical simulation cases had 112 responses across 14 different cases. Almost all residents (99.1%) agreed or strongly agreed that the scenarios were relevant to real-world clinical practice (Table 3).

Measured outcomes demonstrated that 94.7% of participants reported improvement in clinical diagnosis, 93.8% in acute clinical management, and 95.5% in communication and teamwork skills (Table 3). Structured debriefings were consistently cited as a key contributor to learning. Residents noted that these debriefs facilitated clinical reflection and reinforced clinical reasoning.

Learner feedback

Residents provided qualitative comments on the curriculum's overall design and implementation. Positive feedback highlighted the value of hands-on practice, small group format, and skilled facilitation. Representative comments included: "Incredibly useful! Would definitely appreciate more of these sessions," "Very helpful to have hands-on experience," “I wish we could do this more,” and "I like the small group setting and breakdown of proper technique. Staff was very friendly and helpful."

Constructive feedback focused on three main themes: clarification of available resources and procedures, timing consistency between scenarios and debriefs, and optimal scheduling. Specific comments included requests for "clarification who can be called, what procedures can be done," "stricter adherence to timing of scenarios and feedback portions," and suggestions to schedule clinical scenarios before their intensive care unit (ICU) rotations to enhance preparedness.

Faculty assessment of curriculum impact

To further assess the downstream effects of the curriculum, five pulmonary and critical care attending physicians were surveyed at the end of the academic year. These faculty members routinely supervise residents in high-acuity settings, including intensive care and procedural rotations, including pulmonary consults.

A total of 80% of faculty respondents rated PGY-1 resident triaging skills as either “significantly better” or “slightly better” compared to previous years. A total of 60% perceived procedural skills as improved, while all respondents (100%) indicated improvement in POCUS proficiency (Table 4). When asked whether the simulation curriculum contributed to these observed improvements, 80% agreed or strongly agreed regarding its impact on triage abilities, 60% on procedural performance, and 100% on POCUS skill development (Table 5).

**Table 4:** Attending assessment of curriculum effect on intern skills (N = 5). CVC: central venous catheterization; POCUS: point-of-care ultrasound.

Survey question	“Significantly better”	“Slightly better”	“About the same”	“Slightly worse”	“Significantly worse”	Likert scale ± standard deviation
	5	4	3	2	1	(1-5)
Compared to last year, how would you rate this year's intern triaging skills? Consider effectiveness, efficiency, and clinical judgement.	20.0% (1/5)	60.0% (3/5)	20.0% (1/5)	0.0% (0/5)	0.0% (0/5)	4.00 ± 0.58
Compared to last year, how would you rate this year’s interns’ procedural skills? (CVC, thoracentesis, paracentesis, and intubation)	20.0% (1/5)	40.0% (2/5)	40.0% (2/5)	0.0% (0/5)	0.0% (0/5)	3.80 ± 0.75
Compared to last year, how would you rate this year's intern POCUS skills? (Cardiac, lung, vascular, and abdominal ultrasound)	80.0% (4/5)	20.0% (1/5)	0.0% (0/5)	0.0% (0/5)	0.0% (0/5)	4.80 ± 0.40

**Table 5:** Attending perceptions of curriculum’s contribution to intern skill (N = 5). IM: internal medicine; POCUS: point-of-care ultrasound.

Survey question	"Strongly agree"	"Agree"	"Neutral"	"Disagree"	"Strongly disagree"	Likert scale ± standard deviation
	5	4	3	2	1	(1-5)
The new IM simulation curriculum contributed to the observed change in intern triaging skills.	40.0% (2/5)	40.0% (2/5)	20.0% (1/5)	0.0% (0/5)	0.0% (0/5)	4.20 ± 0.75
The new IM simulation curriculum contributed to the observed change in intern procedural skills.	40.0% (2/5)	20.0% (1/5)	40.0% (2/5)	0.0% (0/5)	0.0% (0/5)	4.00 ± 0.89
The new IM simulation curriculum contributed to the observed change in intern POCUS skills.	60.0% (3/5)	40.0% (2/5)	0.0% (0/5)	0.0% (0/5)	0.0% (0/5)	4.60 ± 0.49

These findings suggest that the curriculum not only enhanced resident confidence but also translated into observable improvements in clinical performance as perceived by supervising faculty.

## Discussion

Although many programs employ simulation as an adjunct, few have integrated it as a central element of their educational schedule [2,18-21]. The same was true of our program, which, despite access to a well-equipped simulation center, had previously used the lab only for orientation procedure entrustment, annual skills assessments, and occasional POCUS practice. Our new longitudinal and multimodal simulation-based curriculum represents a meaningful departure from this traditional approach by replacing 50 lecture-based noon conferences with hands-on simulation sessions.

One of the key innovations of this curriculum is its comprehensive design. Rather than focusing exclusively on procedural training or isolated emergency scenarios, this design integrates multiple domains of clinical competency, including POCUS, procedural skills, and acute clinical triaging skills. This deviates from prior studies that evaluate and prove individual simulation-based learning activities are effective [18,22-24]. This comprehensive design allowed for reinforcement of multiple competencies and mirrored real-world clinical complexity.

Another distinctive feature was the intentional inclusion of peer observation. By alternating between active and observing roles, residents were exposed to multiple management strategies and had the opportunity to reflect on clinical decision-making in real time. Peer observation in simulation is less often utilized compared to active participation, and therefore studies on the efficacy of peer observation are limited; however, this is also an effective tool when thoughtfully implemented [25,26]. The role of debriefing in simulation has also been shown to be a crucial component that makes this learning method effective, and, therefore, we ensured that faculty leading the debrief were appropriately trained beforehand [27]. Coupled with structured faculty-led debriefings, this combination created an environment conducive to reflective learning and cognitive unloading and prompted skill retention.

In addition, the curriculum was developed and implemented through a multidisciplinary team involving internal medicine chief residents, internal medicine faculty members, simulationists, and nurses. This collaborative structure not only ensured high-fidelity, clinically relevant scenarios but also modeled the interprofessional teamwork essential in acute care settings.

Beyond these structural innovations, feedback from learners validated core design elements and revealed implementation challenges. Residents' emphasis on hands-on practice, small group format, and requests for increased frequency align with established principles of experiential learning and learner preference for active, simulation-based learning over traditional lectures [3,28]. Constructive feedback regarding resource clarification, timing consistency, and optimal scheduling reflected common implementation challenges in simulation curricula. Notably, residents' suggestion to schedule clinical scenarios before ICU rotations aligns with "just-in-time" training principles, which have been shown to maximize knowledge transfer when learning occurs proximal to clinical application [29]. In response to feedback, the pre-brief was adjusted to provide more detailed information about available resources, and efforts were made to standardize session timing. However, variability persisted due to case heterogeneity and resource constraints, including simulation lab availability and facilitator schedules.

Limitations

While the curriculum demonstrated positive outcomes, several limitations must be acknowledged. First, because it was developed in response to interns' requests for more simulation-based learning, there is an inherent bias toward favorable feedback. Additionally, the primary assessment relied on self-reported post-session surveys, which are subject to response bias and may not accurately reflect actual skill acquisition or clinical performance. Self-reported confidence does not necessarily correlate with objective competence, and learners may overestimate their abilities following positive experiences. Objective measures, such as direct observation in clinical settings or simulation-based competency checklists, were also not utilized and represent an important area for future study. The absence of these measures, combined with reliance on self-reported data, limits the ability to determine whether confidence gains translate into measurable improvements in clinical performance. As a single-group descriptive study without inferential analyses, our results should be interpreted as learner perceptions rather than statistically significant performance changes.

Second, because this was an early-phase implementation study, we did not include a pre-intervention baseline or control group receiving traditional lecture-based training. As such, improvements in learner confidence and performance cannot be attributed solely to the simulation-based curriculum. Observed changes may reflect natural skill development over time, concurrent clinical experiences, or other educational activities rather than the direct impact of our curriculum.

Third, although faculty feedback suggested perceived improvements in clinical performance, the small number of attending respondents (N = 5) limits the generalizability of these observations. With such a limited sample, individual faculty biases or variations in supervision style may have disproportionately influenced results, and the findings may not represent the broader faculty perspectives. Similarly, the curriculum was implemented at a single institution with a relatively small sample size of residents, which may limit broader applicability. Additionally, variability in case complexity and facilitator debriefing styles may have influenced the learning experience across different sessions, as debriefing quality and approach are known to significantly impact simulation-based learning outcomes [27]. This variability limits our ability to attribute outcomes to specific curricular elements and complicates replication at other institutions.

Fourth, the curriculum was restricted to PGY-1 residents, which may limit the applicability of findings to upper-level residents with different baseline competencies and learning needs. This omission was intentional and due to limitations of resource availability, including access to the simulation lab, availability of faculty facilitators, and simulation staff.

Finally, although residents expressed a desire for more simulation-based training, scheduling constraints prevented expansion beyond the 50 sessions integrated into the academic calendar. Future iterations of the curriculum may benefit from increased frequency, spaced repetition, and longitudinal tracking of clinical skill retention to assess sustained impact.

Future directions

Continued evaluation is needed to measure long-term knowledge retention, observe clinical application at the bedside, and compare outcomes with traditional lecture-based education using prospective baseline assessments, validated outcome measures, and comparison groups. Future studies should incorporate these elements to more objectively determine the educational impact of this curriculum. Planned program enhancements include increasing session frequency, integrating spaced-repetition cycles, and implementing longitudinal assessments to monitor skill decay and reinforcement. We also aim to expand the curriculum to PGY-2 and PGY-3 residents with differentiated objectives and more complex scenarios, contingent on resource availability. Furthermore, we plan to incorporate interprofessional simulations with an emphasis on communication and team dynamics, which will further widen educational impact and more closely align with the Accreditation Council for Graduate Medical Education (ACGME) core competencies [30]. These steps will support scalability and establish the broader applicability of simulation-based education in internal medicine training.

## Conclusions

Our simulation-based curriculum effectively replaced traditional lecture content with experiential learning of high impact. Residents reported increased confidence in procedural skills, POCUS, and clinical triage, while faculty independently observed improvements in clinical performance compared to prior cohorts. Pulmonary and critical care attendings further validated the success of the curriculum. The curriculum's multimodal design, encompassing technical skills, ultrasound competencies, and acute care decision-making, addressed multiple domains of clinical competency within a structured, reproducible framework.

This model demonstrates that comprehensive simulation-based education can be successfully embedded within existing residency schedules without requiring additional protected time. By integrating POCUS, procedures, and clinical simulation into a longitudinal curriculum, programs can strengthen experiential learning and enhance resident preparedness for high-acuity clinical scenarios. This scalable, generalizable approach offers a practical framework for internal medicine programs seeking to modernize training through active, learner-centered education.
